# Ovarian Mature and Immature Teratomas in Monozygotic Twins: A Case Report of Simultaneous Presentation

**DOI:** 10.1155/2017/5810515

**Published:** 2017-02-15

**Authors:** Daisuke Shigemi, Naoki Kawai, Toshiyuki Takeshita

**Affiliations:** ^1^Department of Obstetrics and Gynecology, Nippon Medical School, Tokyo, Japan; ^2^Department of Obstetrics and Gynecology, Tokyo Rinkai Hospital, Tokyo, Japan

## Abstract

Mature cystic teratoma is one of the most common kinds of ovarian tumor, and immature teratoma is a rare tumor, representing less than 1% of all ovarian teratomas. Although there are some reports about familial occurrences of ovarian tumors, literature concerning the clinical cases of monozygotic twins is rare. We report the 5-year clinical courses of a 12-year-old Japanese girl with a recurrent bilateral ovarian mature teratoma and her monozygotic twin with a unilateral ovarian mature teratoma and contralateral ovarian immature teratoma. This is the first clinical report on mature and immature teratomas of the ovary in monozygotic twins. Our cases support future clarification of the molecular mechanism and pathogenesis of teratoma.

## 1. Introduction

Benign cystic teratoma of the ovary is the most common type of ovarian neoplasm. By contrast, an immature teratoma is a rare tumor, representing less than 1% of all ovarian teratomas and 1% of all ovarian cancers [1]. Although there are some reports about familial occurrences of ovarian tumors, literature concerning the clinical cases of monozygotic twins is rare. We report the cases of a bilateral and repeated ovarian benign teratoma and an immature teratoma that appeared simultaneously in two monozygotic twins.

## 2. Case Presentation

### 2.1. Case  1

**Figure 1:**
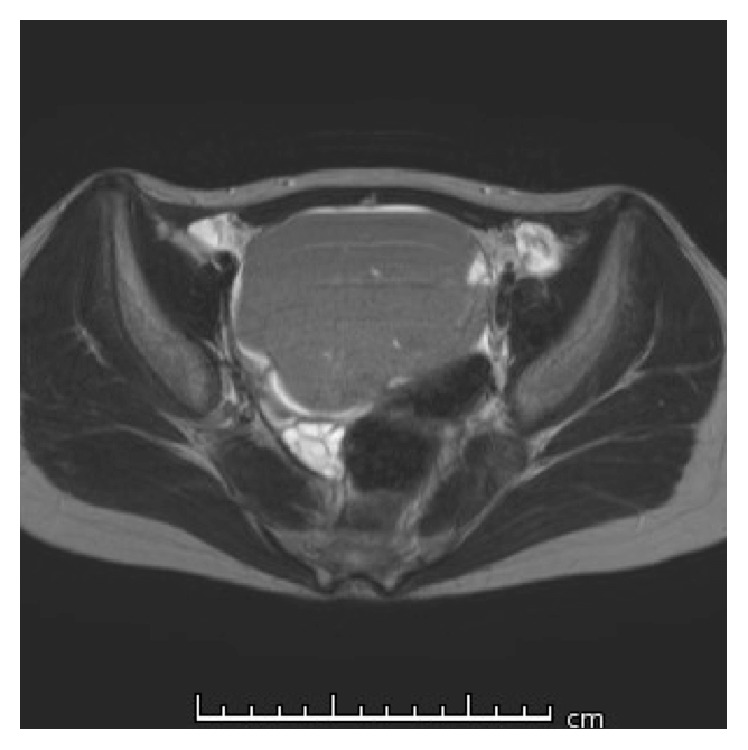
Pelvic magnetic resonance imaging (T2-weighted image) before the first surgery of Case  1. An intrapelvic tumor (9.2 × 7.6 × 7.5 cm in diameter) with high signal intensity is detected. The right ovary is normal.

A 12-year-old Japanese girl, gravida 0 para 0, visited our gynecologic outpatient department for mild lower abdominal pain. Her history was noncontributory, and she had no remarkable family history. A pelvic examination was avoided since she was a virgin. A transabdominal ultrasound test detected a large left ovarian cyst, measuring 9.9 cm × 6.6 cm with calcification inside it, with no vascularity. Her uterus and right adnexa were normal. Magnetic resonance imaging (MRI) of the pelvis revealed a left ovarian tumor (9.2 × 7.6 × 7.5 cm in diameter) with high signal intensity that was capsulated with small amount of fluid in the Douglas pouch (Figure 1). The right ovary was normal. Laboratory examination showed that the level of cancer antigen (CA) 125 was 36.8 U/mL (normal range < 35 U/mL), and no other tumor marker levels were elevated. She underwent laparotomy later. She was also diagnosed with left ovarian tumor without rupture and torsion. A left cystectomy was performed, and no surgical complications occurred. Microscopic and histopathological examination of the cystic mass revealed that it was a benign teratoma emerging from the left ovary.

After discharge, the patient was followed every third month. Fifteen months after the first surgery, a right ovarian cystic mass was detected with an elevated CA125 level (35.6 U/mL), and she presented to the emergency room because of severe lower abdominal pain with acute onset. A right ovarian cyst with torsion was suspected. She underwent laparotomy again, and right salpingooophorectomy was then performed because of twisted and black-and-blue ovarian findings. The patient recovered well after the surgery, and histological analysis of the ovarian cyst revealed that it was a benign teratoma with infarcted and necrotic tissue.

We resumed regular follow-up. Twenty months after the second surgery, a left ovarian cystic tumor (4 cm in size) was detected without any symptoms. Regular pelvic examination was continued, and an enlarged ovarian cystic mass (6.6 cm in size) was diagnosed, with an elevated squamous cell carcinoma (SCC) antigen level (2.0 U/mL; the normal range is less than 1.0 U/mL) on hematological investigation. A third laparotomy was performed, and the left ovarian cystic tumor was resected. The histological diagnosis was a benign teratoma. After 13 months, the patient continued to do well and her blood SCC antigen level was normalized.

### 2.2. Case  2

A 12-year-old Japanese girl, gravida 0 para 0, who was the monozygotic twin sister of the patient in Case  1, visited our gynecologic department for ovarian screening 3 months after the first operation of Case  1. Transrectal echography revealed bilateral ovarian cystic tumors with calcified structures. Tumor marker levels (CA125, SCC, CEA, and CA19-9) were normal. The bilateral ovarian cystic walls were extracted, and normal ovarian tissues were preserved by laparotomy. Pathological analysis of the cyst wall disclosed that there were benign teratomas in both ovaries.

**Figure 2:**
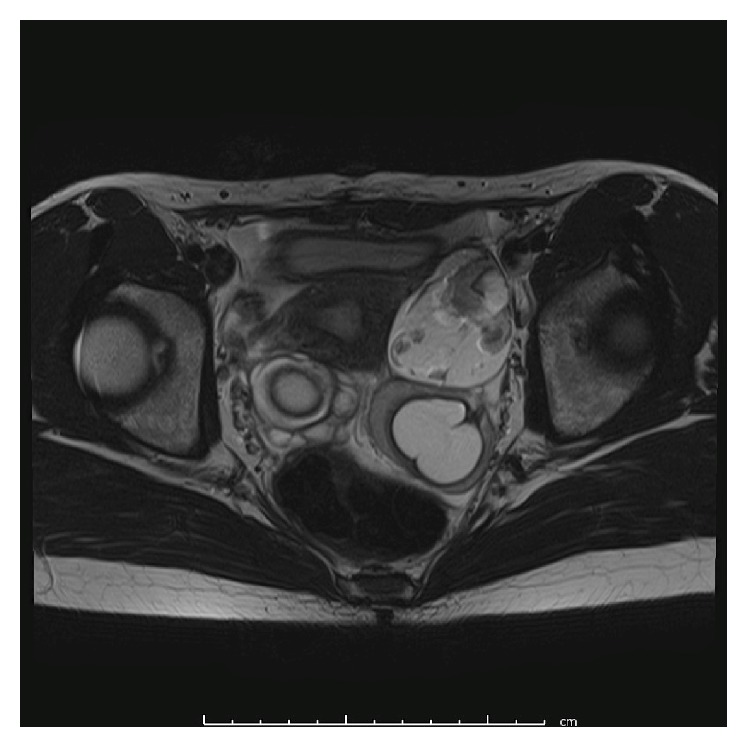
Pelvic magnetic resonance imaging (T2-weighted image) before the second surgery of Case  2. Bilateral ovarian tumors are detected (right side: 4.4 cm, left side: 8.0 cm in diameter).

Sixteen months after the first surgery, bilateral ovarian tumors were detected with echogram and pelvic MRI (right side: 4.4 cm, left side: 8.0 cm in diameter) during her regular check-up (Figure 2). The ovarian tumors were accompanied by a solid part and high echoic part inside. Blood examination of tumor markers revealed a mild, elevated CA125 (37.5 U/mL). Bilateral cystectomy was performed 20 months after the first surgery. Examination of the other pelvic and abdominal organs found no disseminated and metastatic lesions in the surgery. There were no swollen pelvic lymph nodes. The histological diagnosis was an immature teratoma of grade 1 with International Federation of Gynecology and Obstetrics stage I in the right ovary and a benign mature teratoma in the left ovary. The girl's postoperative condition was good, and she was discharged without any complications. An additional surgery and chemotherapy were not planned according to a deliberated discussion with her family.

**Figure 3:**
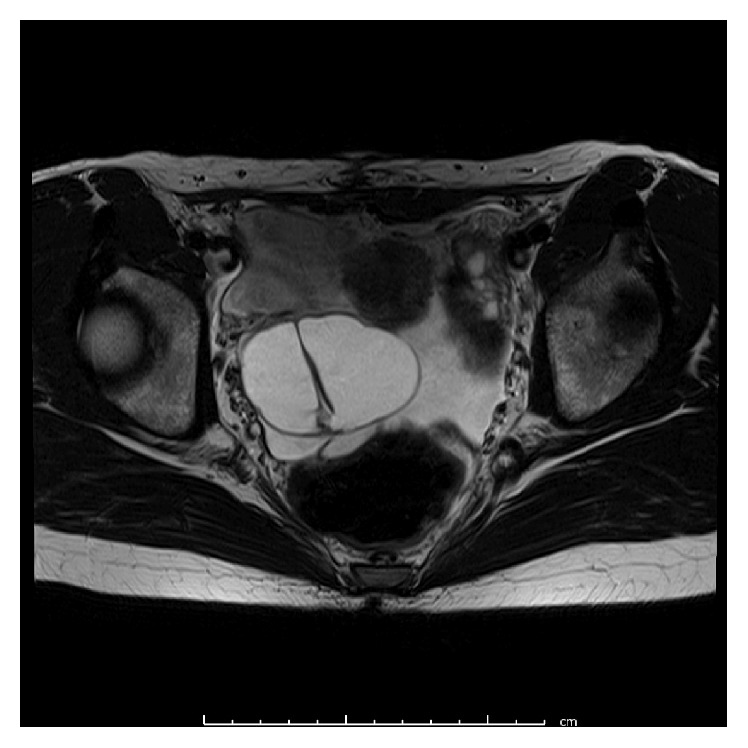
Pelvic magnetic resonance imaging (T2-weighted image) before the third surgery of Case  2. Right ovarian tumors are detected (multiple cystic, 6.3 × 4.9 cm in diameter).

**Figure 4:**
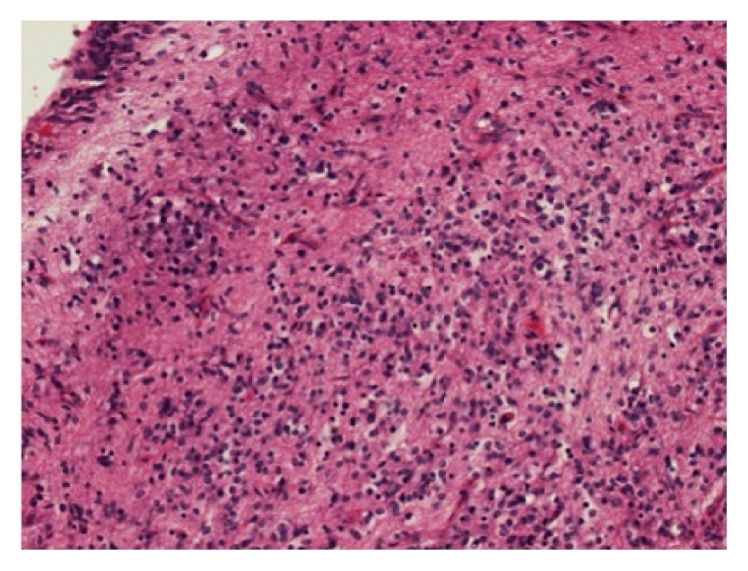
Histological section showing immature teratoma composed of small immature neurogenic cells (hematoxylin and eosin, 100x magnification).

**Figure 5:**
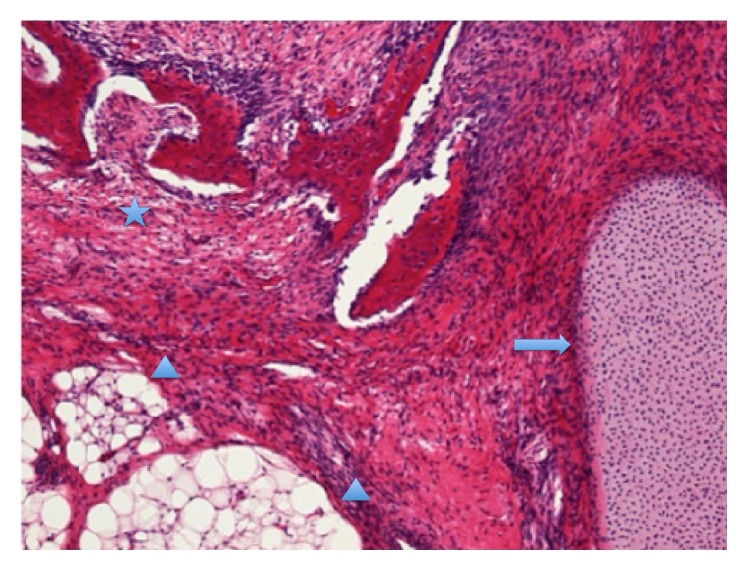
Histological section showing the immature teratoma with immature tissue of the bone (asterisk), cartilage (arrow), and islands of adipose tissue (arrowhead) (hematoxylin and eosin, 100x magnification).

After being discharged, she continued to be followed every other month. Four months after the last surgery, a right ovarian cystic mass (4 cm in size) was detected, and it enlarged to 6 cm two months later (Figure 3). The blood CA125 level (32.3 U/mL) was in the normal range. Therefore, a third laparotomy was performed 7 months after the second surgery. The right adnexa, appendix, and greater omentum were resected. Examination of the other pelvic and abdominal organs showed that they did not have disseminated and metastatic lesions. There were no swollen pelvic lymph nodes. The histological diagnosis was an immature teratoma of grade 1 in the right ovary, and metastatic carcinoma was not detected in the other extracted organs (Figures 4 and 5). After 30 months, the patient continued to do well and there was neither tumor recurrence nor pelvic lymphadenopathy. Levels of tumor markers, including CA125, were within normal levels.

Approval of the institutional study board and informed consent of these patients were obtained.

## 3. Discussion

Mature cystic teratoma is one of the most common kinds of ovarian tumor, with a frequency of approximately 20% [2]. Torsion of the ovary is a major complication, and rupture of the cyst may also occur. Immature teratoma is a rare tumor, representing less than 1% of all ovarian teratomas, 1% of all ovarian cancers, and 35.6% of malignant ovarian germ cell tumors [1]. They are most common in the first two decades of life [1]. However, data on the management and treatment of immature teratoma are limited because of its rarity. According to a recent study that assessed 27 patients with immature teratoma, 82% had an International Federation of Gynecology and Obstetrics stage I, 11% had stage II, and 7% had stage III disease [3].

Ovarian teratoma is considered an acquired neoplastic disease; therefore, familial incidence has been reported in only a few papers. Only three studies have reported teratoma of the ovary in twins [4–6], only one of which described monozygotic twins [4]. Some previous studies reported endometrioma, borderline tumors, and carcinoma of the ovary in twins, but studies on mature cystic teratomas and immature teratomas are relatively rare.

Teratoma contains elements of the ectodermal component in most tumors, and the cause has not been identified. There are two main hypotheses for its etiology. The first is based on totipotent cells, which are segregated in the development of the morula. Subsequently, derivates of all germinal cell layers occur, and they are similar to those in the contiguous tissues. The second hypothesis, which is the most accepted theory, supposes that an unknown trigger stimulates asexual development or parthenogenesis [6]. Parthenogenesis mentions development of embryo without the male gamete. Chromosome banding studies and enzyme polymorphisms helped the pathogenic research of gonadal teratomas from 1970s. Linder et al. suggested that benign cystic teratomas of the ovary are derived from parthenogenetically activated ovarian oocytes after meiosis I, but before meiosis II [7, 8]. Additional analysis indicates that the DNA content and karyotype (46,XX) of mature cystic teratomas of the ovary are usually homozygous and it strongly supports Linder's hypothesis of a germ cell origin [9]. Since parthenogenetic activation of meiosis I oocytes takes place in the ovarian follicles, there are probably other factors which contribute to the development and growth of the parthenote. It was shown that these abnormal embryos may develop to form the bizarre messes which consist of a variety of differentiated cell and tissue types, a unique feature of the ovarian teratomas [10].

Additionally, a disorder of “genomic imprinting” is considered as an important mechanism in pathogenesis of ovarian teratomas. Genomic imprinting is the phenomenon that the paternal and maternal sets of chromosomes have different functionality in mammals, due to parental-specific epigenetic modification of the genome. Thus, the allelic expression of an imprinted gene depends upon whether it resided in a male or a female in the previous generation. Imprinted expression can also vary between tissues, developmental stages, and species [11]. The presence of genomic imprinting in mammals has considerable medical, societal, and intellectual implications in terms of the clinical management of genetic traits and diseases, the capacity to control human and animal breeding by assisted reproductive technologies, and the progress of biotechnology and postgenomic medical research [12]. According to previous research, parthenogenesis in teratomas of the ovary can be considered as control by genomic imprinting from mother. Thus, although the etiology and mechanism of the simultaneous occurrence of ovarian teratomas in monozygotic twins are not proven, they could be explained by these theories.

Some malignant tumors in other regions, such as gastric cancer, breast cancer, or neuroblastoma, have been reported to present simultaneously in monozygotic twins. The etiology of malignant cases has been debated: is the disease a simultaneous occurrence in both twins, or is it because of metastatic spread via placental vascular anastomoses in utero from one twin with a congenital disease to the second twin? By contrast, it is difficult to speculate about metastatic spread in the twins in the present cases, because one had a benign, mature teratoma and the other had a malignant, immature teratoma. Linder and Power have reported that monozygotic twins have a higher chance of malignant suffering than fraternal twins do [7]. According to the latest prospective study of over 80,000 monozygotic twins, 38% of monozygotic twins were diagnosed with the same cancer type, and significant heritability (39%) was observed for ovarian cancers [8]. We continue to closely follow up the patient described in Case  1, considering the chance of genetic risk of malignant presentation.

Currently, information describing the development of teratoma of the ovary in twins globally is scarce. This is the first clinical report on mature teratoma and immature teratoma of the ovary in two monozygotic twins. Our cases are only a small part of the reports on ovarian mature teratoma and immature teratoma in monozygotic twins, but our report supports future clarification of the molecular mechanism and pathogenesis including the parthenogenesis with genomic imprinting of ovarian teratomas.
